# Bilateral increase in expression and concentration of tachykinin in a unilateral rabbit muscle overuse model that leads to myositis

**DOI:** 10.1186/1471-2474-14-134

**Published:** 2013-04-12

**Authors:** Yafeng Song, Per S Stål, Ji-Guo Yu, Sture Forsgren

**Affiliations:** 1 Department of Integrative Medical Biology, Section for Anatomy, Umeå University, Umeå, Sweden; 2 Department of Surgical and Perioperative Sciences, Sports Medicine, Umeå University, Umeå, Sweden

**Keywords:** Muscle, Triceps surae, Muscle overuse, Myositis, Inflammation, Tachykinin, Substance P

## Abstract

**Background:**

Tachykinins can have pro-inflammatory as well as healing effects during tissue reorganization and inflammation. Recent studies report an up-regulation in the expression of the substance P (SP)-preferred receptor, the neurokinin-1 receptor, in marked muscle inflammation (myositis). There is, however, only very little information on the expression patterns and levels of tachykinins in this situation.

**Methods:**

The tachykinin system was analyzed using a rabbit experimental model of muscle overuse, whereby unilateral muscle exercise in combination with electrical stimulation led to muscle derangement and myositis in the triceps surae muscle (experimental length 1–6 weeks). Evaluations were made for both parts of the muscle (soleus and gastrocnemius muscles) in experimental and non-experimental (contralateral) sides. Morphologic evaluation, immunohistochemistry, *in situ* hybridization and enzyme immunoassay (EIA) analyses were applied.

**Results:**

Myositis and muscle derangement occurred focally not only in the experimental side but also in the non-experimental side. In the inflammatory areas (focal myositis areas), there were frequent nerve fibers showing tachykinin-like immunoreactivity and which were parts of nerve fascicles and which were freely dispersed in the tissue. Cells in the inflammatory infiltrates showed tachykinin-like immunoreactivity and tachykinin mRNA expression. Specific immunoreactivity and mRNA expression were noted in blood vessel walls of both sides, especially in focally affected areas. With increasing experimental length, we observed an increase in the degree of immunoreactivity in the vessel walls. The EIA analyses showed that the concentration of tachykinin in the tissue on both sides increased in a time-dependent manner. There was a statistical correlation in the concentration of tachykinin and the level of tachykinin immunoreactivity in the blood vessel walls between experimental and non-experimental sides.

**Conclusions:**

The observations show an up-regulation of the tachykinin system bilaterally during muscle derangement/myositis in response to pronounced unilateral muscle overuse. This up-regulation occurred in inflammatory areas and was related not only to increased tachykinin innervation but also to tachykinin expression in blood vessel walls and inflammatory cells. Importantly, the tachykinin system appears to be an important factor not only ipsilaterally but also contralaterally in these processes.

## Background

The tachykinin system is consituted by the previously well characterized neuropeptides substance P (SP), neurokinin A and neurokinin B, as well as the more recently described endokinins and hemokinins [1-4]. The tachykinins and their structurally related peptides comprise a very large superfamily with a high degree of functional diversity. The discovery of the preprotachykinin gene TAC4 has much increased the number of tachykinins detected to date [2,5]. A new tachykinin gene-related peptide (EK-2) has e.g. been verified for rabbit tissue [2].

Substance P (SP) was the first tachykinin to be discovered and is the tachykinin that is most widely studied. Originally isolated from the brain and gut of the horse [6], SP is derived after post-translational modification of the preprotachykinin A gene and shows preferential binding to the neurokinin-1 receptor (NK-1R) [7]. Tachykinins, including SP, seem to be of great importance in inflammatory [8-10] and trophic and proliferative [11] processes. It is wellknown that SP has proinflammatory effects [12,13].

Muscle injury and inflammation (myositis) can develop in response to marked overuse of skeletal muscles. We have created an animal (rabbit) overuse model, involving the triceps surae muscle, where muscle overuse is established via exercise in combination with electrical stimulation, and which leads to structural changes in the tissue, including muscle fiber necrosis and myositis [14]. Interestingly, in our previous study, we identified that myositis and structural alterations developed not only in the muscles in the exercised side, but also in the muscles in the contralateral, non-exercised side, suggestive of a cross-over effect. The structural changes that occurred were focal; that is, they were noticeable only in certain parts of the muscle and were interpreted to be related to degenerative/regenerative processes occurring in the muscle and to influences on the innervation at these specific sites [14].

To date, there is very little information as to the possible involvement of tachykinins in the muscle tissue in situations with marked muscle derangement/myositis in response to pronounced overuse. What is known concerning tachykinins and affected muscle tissue is that injection of Freund´s adjuvant into the gastrocnemius muscle of rats is followed by an increase in SP-innervation [15]. Hoheisel and collaborators further demonstrated a relationship between neuronal activity and the pattern of SP-immunoreactivity in the rat spinal cord during acute and persistent muscle inflammation [16]. Craniofacial muscle inflammation has also been shown to lead to an increase in the number of SP- and CGRP-immunopositive ganglion neurons innervating the inflamed muscle [17]. Using our rabbit overuse model, we recently noticed up-regulation in the expression of the NK-1R in the inflammatory areas [18].

In our studies on the expression patterns of the NK-1R referred to above, brief observations concerning tachykinin expression patterns and levels were noticed. No information at all was collected concerning the reaction patterns for the blood vessel walls and no evaluations were made at the mRNA level concerning tachykinins. No thorough investigation on the possibly changing tachykinin expressions and levels for the various stages of overuse was made. Thus, it is indeed important to know in detail how the expression and levels of tachykinin are influenced during the processes of marked muscle derangement and myositis.

In the present study, we used our rabbit model of muscle overuse to investigate the importance of tachykinins in the processes of myositis and structural changes that occur in response to marked muscle overuse. We evaluated the ipsilateral and contralateral soleus and gastrocnemius muscles of the triceps surae muscle using immunohistochemistry, *in situ* hybridization and enzyme immunoassay (EIA) analyses. The observations show that there is an increasing involvement of the tachykinin system both ipsilaterally and contralaterally with increasing duration of the experiment. Several structures were involved in the upregulation; the innervation, the inflammatory cells and the blood vessel walls.

## Methods

### Ethics statement

The animal studies have been conducted according to national and international guidelines, including in accordance with EU Directive 2010/63/EU for animal experiments. The study protocol was approved by the local ethical committee at Umeå University (A34/07). A licensed breeder had bred all animals for the sole purpose of being used in animal experiments.

### Animals

A total of 24 New Zealand adult white female rabbits were used in this experiment. The animals weighed approximately 4 kg and had an age ranging from 6–9 months. They were divided into four groups consisting of six animals in each group. The animals of three of the groups were exposed to the experiment procedure on their right leg, as described below. The animals of the fourth group served as controls and did not undergo any experiment at all.

All animals were anaesthetized during the exercise procedure, by means of an intramuscular injection of fentanylfluanison (0.2-0.3 ml/kg) and diazepam (0.2 ml/kg; 5 mg/ml), followed by additional injections of fentanylfluanison (0.1 ml/kg) every 30–45 min during the experimental procedure, in order to maintain anaesthesia. Buprenorphine, 0.01-0.05 mg/kg, was given s.c. postoperatively.

### Experimental design

The purpose of the use of the model was to achieve a situation with marked muscle overuse. In order to achieve this, an apparatus (“kicking machine”), was used, influencing the triceps surae muscle. The procedures were those previously used in studies on the tendon part (the Achilles tendon) of the muscle [19] and conform to those utilized in studies on muscle derangement/myositis [14,18]. The model is originally designed by Backman and collaborators [20] but was used with some modifications. The apparatus is constructed to generate passive flexion and extension of the ankle joint in one of the legs (the right leg). Movements are produced by means of a pneumatic piston. In order to produce further strain on the muscle/tendon of the right leg, electric stimulation via surface electrodes (pediatric electrodes 40 426A, Hewlett Packard, Andover, MA, U.S.A), that gives rise to contraction of the triceps surae muscle, was applied. For further details of the procedures, see [14,18].

The experiment was performed for 2 h every second day**,** for a total period of one, three, and six weeks respectively. Six animals were subjected to the experimental procedure for each of these time periods. It was not clearly obvious that the animals showed markedly amended movements or changed behaviours inbetween the experiment periods.

### Sampling of specimens

One day after the last bout of exercise, the rabbits were sacrificed and the triceps surae muscle was dissected out. From all animals, the muscles of both the right and the left sides were harvested. The tissue samples were immediately taken to the laboratory, and samples corresponding to samples of the distal parts of the soleus and gastrocnemius muscles were further processed for microscopic analyses (morphology, immunohistochemistry and *in situ* hybridization) and for enzyme immunoassay (EIA).

### Processing for morphology and immunohistochemistry

#### Fixation and sectioning

Tissue specimens from all muscles were immediately fixed by immersion overnight at 4°C, in 4% formaldehyde in 0.1 M phosphate buffered solution, pH 7.0. The following steps concerning washing, mounting and freezing were as previously described [14,18]. The specimens were processed for demonstration of routine morphology and for immunohistochemistry and *in situ* hybridization. Other tissue specimens from all muscles were directly mounted and frozen as described above, i.e. processed chemically unfixed, and these were used for delineating tissue morphology. Still other specimens were harvested and used for enzyme immunoassay (EIA) (c.f. below).

The specimens were cut in series of 7–8 μm thick sections for use in immunohistochemistry, using a cryostat before being mounted on chrome-alum gelatine pre-coated slides. Sections of all specimens (chemically fixed as well as unfixed) were processed for morphology via staining for htx & eosin.

#### Immunohistochemical staining for tachykinin

Sections from all the muscle specimens were processed for tachykinin immunolabelling. In order to broaden the picture concerning tachykinin immunoreactivity, a polyclonal as well as a monoclonal antibody was utilized. The sections were initially incubated for 20 min in a 1% solution of Triton X-100 (Kebo lab, Stockholm) in 0.01 M phosphate buffered saline (PBS), pH 7.2, containing 0.1% sodium azide as preservative, and were thereafter rinsed in PBS three times, 5 min each time. The sections were then incubated in 5% normal donkey serum in PBS for 15 min. The sections were thereafter incubated with the primary tachykinin antibody, diluted in PBS [concerning polyclonal tachykinin antibody produced in goat (SP antibody; sc-14104) (for further details, see below)] or PBS with bovine serum albumin (BSA) [concerning the monoclonal tachykinin antibody used and which was produced in rat (8450–0505)] in a humid environment. Incubation was performed for 60 min at 37°C. After incubation with specific antiserum, and after washes in PBS 5 min × 3, another incubation in normal donkey serum followed, after which the sections were incubated with secondary antiserum. As secondary antiserum, fluorescein isothiocyanate (FITC)-conjugated AffiniPure donkey anti-goat IgG (705-095-147) (Jackson ImmunoResearch, PA), diluted 1:100 in PBS, was used for demonstration of reactions for sc-14104. Tetramethylrhodamine isothiocyanate (TRITC)-conjugated AffiniPure donkey anti-rat IgG (712-025-150) (Jackson ImmunoResearch), diluted 1:40 in PBS supplemented with 0.1% BSA, was used for demonstration of reactions for 8450–0505. Incubation with secondary antisera proceded in both cases for 30 min at 37°C. The sections were thereafter washed in PBS and were then mounted in Vectashield Mounting Medium (H-1000) (Vector Laboratories, Burlingame, CA, USA). Examination was carried out in a Zeiss Axioscope 2 plus microscope equipped with an Olympus DP70 digital camera.

In parallel with the staining procedure described above, pretreatment with acid potassium permanganate for 2 min, a procedure found to enhance specific immunofluorescence reactions for certain substances [21], was added in the procedure for stainings. It was performed as an initial step. Both variants of procedures (with or without this pretreatment) gave reliable results but with somewhat different optimal reactions for the various structures. It was therefore found relevant to use both variants.

#### Double staining for tachykinin/white blood cell markers, tachykinin/betaIII-tubulin and tachykinin/S-100beta

With the purpose to identify the cell types for which tachykinin-like immunoreactivity occurred, double-stainings concerning tachykinin/white blood cells were performed. Furthermore, it was found relevant to clarify the nerve-related reactions. Therefore, double-stainings tachykinin/betaIII-tubulin and tachykinin/S-100beta were performed. BetaIII-tubulin is an axonal marker and S-100 is used for depicting the Schwann cells. Due to suitability of antibody type for these stainings, the polyclonal tachykinin antibody produced in goat (sc-14104) was hereby used.

The sections were rinsed in PBS 4×2.5 min, and the sections were then incubated for 20 min in a 1% solution of Triton X-100 (Kebo lab, Stockholm) in 0.01 M PBS, pH 7.2, containing 0. 1% sodium azide as preservative, and were thereafter rinsed in PBS 4×2.5 min. The sections were then incubated in 5% normal donkey serum in PBS for 15 min. The sections were thereafter incubated with sc-14104, diluted in PBS, in a humid environment. Incubation was performed for 60 min at 37°C. After 4×2.5 min washes in PBS, a new incubation in normal donkey serum followed, after which the sections were incubated in fluorescein (FITC)-conjugated AffiniPure donkey anti-goat lgG (705-095-147) (Jackson ImmunoResearch, PA), diluted 1:100 in PBS, for 30 min at 37°C. The sections were then rinsed in PBS 4×2.5 min, and incubated in 5% normal rabbit serum in PBS with BSA for 15 min. After that, the sections were incubated with the other primary antibody to be stained for (all of which were mouse monoclonal antibodies), and which was against white blood cell marker, betaIII-Tubulin or S-100beta, diluted in PBS with BSA, in a humid environment. Incubation was performed for 60 min at 37°C. After incubation with this antiserum and 4×2.5 min washes in PBS, a new incubation in normal rabbit serum followed, after which the sections were incubated in rabbit anti-mouse immunolobulins/TRITC (R0276) (Dako, Denmark). As an alternative procedure, normal donkey serum was used instead of normal rabbit serum, and in these cases, donkey anti-mouse immunoglobulins/TRITC (715-295-150) (Jackson ImmunoResearch) was utilized. Both variants gave similar and reliable results. The secondary antibody was used at a dilution of 1:40 (R0276) or 1:500 (715-295-150), the incubation with secondary antiserum in both cases proceeding for 30 min at 37°C. The sections were thereafter washed in PBS for 4×2.5 min and were then mounted in Vectashield Mounting Medium (H-1000) (Vector Laboratories, Burlingame, CA, USA) or Mounting Medium with DAPI (H-1500) (Vector Laboratories) in order to identify nuclei.

#### Double staining for tachykinin/CD31

It was found relevant to clarify the tachykinin immunoreactivity for blood vessel walls in relation to reactions for CD31. Double staining tachykinin/CD31 was therefore made. The initial staining was made for CD31 and the following staining was for tachykinin. The CD31 staining was performed using visualization with FITC-conjugated donkey anti-mouse IgG (715-095-15) (Jackson ImmunoResearch), dilution used 1:100, and as normal serum, normal donkey serum with PBS in BSA was utilized. For demonstration of tachykinin immunoreaction, Alexa FluorO 568 donkey anti-goat (Invitrogen, dilution 1:300) was used. In this case, normal donkey serum in PBS was used. Mounting was performed in Mounting Medium with DAPI (H-1500, Vector Laboratories).

#### Double staining for the polyclonal/monoclonal tachykinin antibodies

In order to compare the reaction patterns for the two tachykinin antibodies used, double staining was performed.

The initial procedures conformed to the procedures described above concering staining for the polyclonal antibody sc-14104. That included the use of FITC-conjugated AffiniPure donkey anti-goat IgG (705-095-147). The sections were then rinsed in PBS 4×2.5 min, and incubated in 5% normal donkey serum in PBS for 15 min. The sections were thereafter incubated with the monoclonal SP-antibody (8450–0505), diluted 1:50 in PBS with BSA, for 60 min at 37°C. After incubation with this antiserum and 4×2.5 min washes in PBS, a new incubation in normal donkey serum followed, after which the sections were incubated in TRITC-conjugated AffiniPure donkey anti-rat lgG (712-025-150), diluted 1:40 in PBS supplemented with 0.1% BSA, for 30 min at 37°C. The procedures for washing, mounting and microscopy were as described above.

### Antibodies and control stainings

#### Tachykinin antibodies

Two different tachykinin antibodies were used. One was an affinity purified goat polyclonal antibody. It was obtained from Santa Cruz Biotechnology (code: sc-14104). It was used at a dilution of 1:25–1:50. A monoclonal SP antibody was also used. It is produced in rat and was obtained from Biogenesis, Poole, UK (code: 8450–0505). It was used at a dilution of 1:50–1:100.

Sc-14104 is raised against a peptide mapping within an internal region of preprotachykinin 1 of human origin and is recommended for detection of mature SP and all isoforms of the protachykinin 1 precursor of various species [mouse, rat and human origin]. It should here be recalled that, according to “Human Protein Reference Database” and “BLAST-Basic Local Alignment Search Tool”, the protein sequence of rabbit protachykinin 1 shows at least 85% identity with human protachykinin 1. It should also be recalled that neurokinin A is derived from the same gene (the preprotachykinin A gene) as SP (TAC1). The antibody 8450–0505 recognizes the COOH terminal end of SP and has been previously used for immunohistochemical detection of SP in experimental animals and man [22,23].

#### Control stainings concerning tachykinin antibodies

The control stainings performed included the use of SP blocking substance (code: sc14104P; Santa Cruz) (20–50 μg/ml antiserum) and SP peptide (full length SP peptide) from Sigma (S6883) (50 μg/ml antiserum). Ordinary stainings with the tachykinin (SP) antisera were performed in parallel. Other control stainings conformed to stainings when the primary antibodies were excluded (buffer instead of the antibody).

#### White blood cell antibodies

The pattern of tachykinin expression in relation to that of white blood cell markers was examined. For this purpose, double-stainings for tachykinin (SP) vs CD68, eosinophil peroxidase and Tcell/neutrophil marker were made.

A mouse monoclonal anti-human CD68 antibody, (code no: M0814), from DAKOCytomation (Glostrup, Denmark) was used for identification of macrophages. It was used at a dilution of 1:100 in 0.1% BSA in PBS. The antigen for this antibody is a glycosylated transmembrane glycoprotein, which is mainly located in lysosomes.

A mouse anti-rabbit T cell/neutrophil antibody from AbD Serotec (Oxford, UK) (MCA805G), which is an affinity purified mouse monoclonal antibody against a cell surface antigen, and which is reported to be expressed by a sub-set of T-cells, thymocytes, neutrophils and platelets, was furthermore used. It was utilized at a dilution of 1:100 in 0.1% BSA in PBS.

The mouse monoclonal antibody MAB1087 from Chemicon (Temecula, CA, USA), which reacts with human eosinophil peroxidase, was also used. This antibody was used at a dilution of 1:100 in 0.1% BSA in PBS.

#### BetaIII-tubulin, S-100beta and CD31 antibodies

A monoclonal antibody against betaIII-tubulin produced in mouse (T8660) (Sigma-Aldrich, USA) was used in double-labelling studies. It was used at a dilution of 1:500. The antibody specificially recognizes an epitope located on isotype III of beta-tubulin. The supplier reports that there is a reactivity for this epitope for various species. No cross-reactivity are reported for other tubulin isotypes. The antibody has been previously used with success by researchers at the Department [24,25]. A mouse monoclonal antibody against S-100 (beta-subunit) was furthermore utilized (S 2532) (Sigma, New York, NY). It was used at a dilution of 1:500. The antibody recognizes an epitope located on the beta-chain, but not the alpha-chain, of S-100. Cross-reactivity has been observed with S-100beta of various species, including rabbit. An antibody against CD31 was utilized as well. It is a monoclonal antibody produced in mice and was obtained from Dako, Glostrup, Denmark (code M0823). It was used at a dilution of 1:100. It is a marker of the endothelial cells of blood vessel walls and has previously been used with reliable results in our laboratory [19].

### Evaluation of vascular tachykinin-like immunoreactions

It was found relevant to determine the levels of tachykinin-like immunoreactions of blood vessel walls. Evaluations of these levels in all identified arteries/arterioles and veins/venules were therefore made in one section from each specimen (vessels of capillary size were not included). The entire sections were evaluated. The evaluations were based on the immunofluorescence intensity seen after staining with the polyclonal antibody sc-14104 (0= no reaction, 1=weak reaction, 2=moderate reaction, 3=high level of reaction). The fluorescence intensity was thus the factor that was evaluated for and a highly fluorescent vein was given the same score as a highly fluorescent arteriole, although the arterial wall was larger. The evaluation was performed blinded. The degrees of expression for all identified vessels in the section were first separately defined whereafter the mean fluorescence intensity for all the blood vessels in the section was defined. Thereafter, the mean intensity for the entire animal group was defined.

### Evaluation of vessel density

To get further information on the vascular changes that possibly occured, overall blood vessel density was calculated. Vessel density (VD) was calculated as the total number of identified blood vessels (arteries/arterioles and veins/venules; capillaries being excluded) per mm^2^ muscle cross-section area in sections from all specimens, after staining for Htx-eosin and for tachykinin-like immunoreaction.

### Processing for *in situ* hybridization

A digoxigenin (DIG)-hyperlabeled oligonucleotide probe (ssDNA) for detection of tachykinin (SP) mRNA was used. In total, 11 different specimens were evaluated concerning *in situ* hybridization.

The sequence of the antisense probe was that of rabbit substance P, CCGTTTGCCCATTAATCCAAAGAACTGCTGAGGCTTGGGTCTCCG (45 bp) (GeneDetect, Auckland, New Zealand). It is well-known that cross-reactions between different tachykinins can occur. Based on previous descriptions that hemokinin-1 is frequently expressed in non-neuronal cells, notably inflammatory cells [26], it was found particularly relevant to evaluate if there might be cross-reactivity with hemokinin-1. “BLAST-Basic Local Alignment Search Tool” test concerning the probe we used was therefore performed. It showed that the probe will not detect rabbit hemokinin-1 (TAC4).

The procedures were performed according an established protocol [27], using an alkaline phosphatase-labeled anti-DIG antibody (GeneDetect) for detection [28,29]. The specimens used for *in situ* hybridization were from the 1 week group (2 samples; one from exercised side, one from contralateral side), the 3 week group (2 samples; one from each side) and the 6 week group (7 samples; 2 from exercised side, 5 from contralateral side). The choosing of samples was based on previous knowledge that a myositis is not observable in the controls and that the most marked myositis and structural affection is seen in the 6 week group [14). A limited number of samples were thus examined via *in situ* hybridization, but we consider that the samples that were utilized were representative samples.

The frozen tissue samples were cut into 10 μm thick fresh cryosections using a cryostat (with a knife washed in 70% EtOH in DEPC-H_2_O) and mounted onto Super Frost Plus slides (nr.041200, Menzel Gläser). After that, the sections were air-dried at room temperature (RT) for 30 min and thereafter fixed in 4% paraformaldehyde (PFA) in 0.1 M phosphate buffer (diluted in DEPC-H2O) (the PFA solution was first passed through a 0.45 μm sterile filter) for 1 h at RT. For details with respect to contents in hybridization solution and all the steps in the staining procedure, see [28,29]. The concentration of the antisense probe was 50 ng in 15 μl hybridization solution, diluted in buffer. The sections were finally mounted in Pertex mounting medium.

The corresponding sense DIG-hyperlabeled ssDNA probe was used as a negative control. As positive control probe, a β-actin probe (GD5000-OP) was used (GeneDetect, New Zealand).

### Processing for enzyme immunoassay (EIA)

#### Tissue homogenization

Tissue samples from all muscles were directly after being weighed frozen in liquid nitrogen. The weights were adjusted to approximately be 30 mg in each case. After being frozen, the samples were homogenised, by mechanical homogenization using Precellys 24 tissue homogenizer (Bertin Technologies, Saint Quentin en Yvelines Cedex, France), in a prepared 100 mM Tris–HCl buffer, pH 7.0, containing 1 M NaCl, 2% BSA, 4 mM EDTA, 0.2% Triton X-100 (pH 7), 0.02% Na-azide and the protease inhibitors Pepstatin A (0.1 μg/ml), Aprotinin (5 μg/ml), Antipain (0.5 μg/ml), Benzamidin (167 μg/ml) and PMSF (5.2 μg/ml). All protease inhibitors were purchased from Sigma, Germany. Tissue and buffer were mixed in a 1:20 relation. The procedure was performed on ice. Directly after the homogenisation procedure, the tissue samples were centrifuged in +4°C, 13 000 g, for 15 min. The supernatant was then transferred to a new Eppendorf tube and stored at −80°C.

#### EIA procedure

The concentration of tachykinin in the muscle samples from all the animals was evaluated using commercially available enzyme immunoassay SP kits (Phoenix Pharmaceuticals, Burlingame, CA, USA). The sequence of the peptide detected conforms to the full-length of SP. 100% cross-reactivity is reported also for SP 2–11 – SP 5–11. Less than 0.01% cross-reactivity is reported for SP 7–11, neuropeptide K and neurokinin A and 0% for neurokinin B. The assays were performed in accordance with the instructions from the manufacturer. In order to obtain comparable results between different plates, reference samples were included in the various analyses. The levels of SP were normalized to the weights of the tissue samples, i.e. the values are expressed as pg/mg tissue.

### Statistical analysis

Mean values and standard deviation were calculated for descriptive statistics. A two way analysis of variance test (ANOVA) was used for analysis of differences in mean values within and between the different experimental groups and the controls. The analysis of normality in the distribution of the data showed no indications of a skewed distribution within each group. A Pearson r test was used to measure correlations between the variables. All the statistical analysis was performed by using the statistical software SAS/STAT vers, 9.2 (SAS Institute Inc, USA). A p-value <0.05 was considered to be significant.

## Results

### Morphology

**Figure 1:**
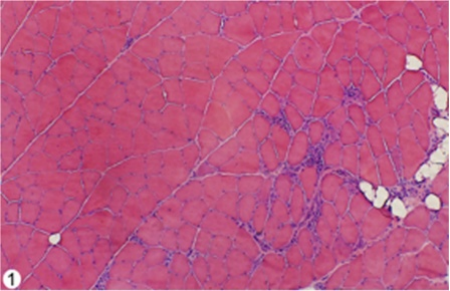
**Overview showing parts of a soleus muscle specimen.** Specimen from the 6w group, exercised side. There is a heterogenity in the tissue. An affected morphology is seen in parts of the tissue, particularly in the form of an inflammatory infiltrate (right part). The left part shows a rather normal morphology. Fat deposits are seen in association with the inflammatory infiltrate. Magnification x50.

**Figure 2:**
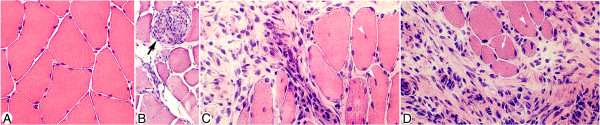
**Occurrence of morphological changes in focal areas of the muscle tissue.** Parts of sections of soleus muscles from a control animal (**A**), the 1w (**B**) and 6w (**C**) groups (exercised side), and the 6w group (**D**) (non-exercised side), stained with hematoxylin & eosin (H&E). Note that there is a very pronounced inflammatory infiltration and a marked presence of loose connective tissue in (**C**, **D**) as compared to in (**A**). Note also that there are pronounced morphological differences between (**A**) and (**B**-**D**); there are variations in fiber size, and occurrence of fiber splitting (white arrow, **D**), necrotic fiber (black arrow, **B**) and frequent internal nuclei (white arrowheads, **C**, **D**) in the sections of the experimental animals. Magnification x200 (**A**, **C**, **D**), x100 (**B**).

In accordance with the observations made in a previous study [14], a muscle inflammation (myositis) was seen in certain parts of the specimens of the soleus and gastrocnemius muscles, especially in the 3 and 6w groups (Figure 1). There was thus a focal presence of inflammatory infiltrates containing groups of white blood cells (“focal myositis”) (Figure 1). Presence of structural changes such as muscle fiber necrosis (muscle fibers being completely filled with infiltrated cells) (Figure 2B) and internal nuclei and fiber splitting (Figure 2C,D) were also seen in these focal areas. There were frequent small-sized blood vessels within these areas and in the areas that were located close by (see further below). A tendency for focal myositis was also observed in the 1w group. Importantly, we noted that the various morphologic changes and inflammatory infiltrations were found not only in muscle specimens of the exercised side (Figure 2B,C), but also in those of the non-exercised contralateral side (Figure 2D).

### *In situ* hybridization

**Figure 3:**
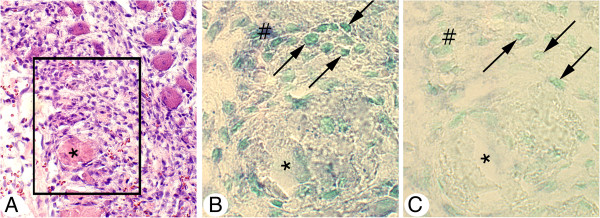
**Expression of tachykinin mRNA in affected muscle tissue as seen via *in situ *hybridization.** Serial sections of a gastrocnemius muscle specimen from the 1w group, exercised side, showing parts of the specimen in low magnification (**A**) (H&E). The framed region in (**A**) corresponds to the regions shown in (**B**, **C**). Note the presence of a marked inflammatory infiltrate and affected muscle fibers in (**A**). In (**B**), expression of tachykinin mRNA in cells of the inflammatory infiltrate (darks spots) (arrows) is seen (antisense staining). There is no reaction in the sense control (**C**, arrows at cells). Asterisk at corresponding muscle fiber location. # indicates similar regions in (**B**, **C**) (artifact in **B**). Magnification x200 (**A**), x315 (**B**, **C**).

**Figure 4:**
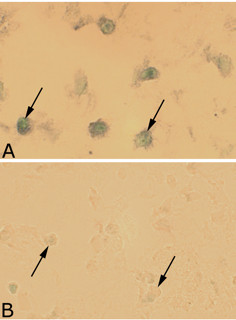
***In situ *hybridization detection of tachykinin mRNA in cells of inflammatory infiltrate.** Sections of a gastrocnemius muscle specimen of the 1w group, exercised side. *In situ* hybridization (**A**: antisense staining, **B**: sense staining). (**A**) and (**B**) are from the same region of the specimen. There is a presence of tachykinin mRNA reactions in the form of black granular reactions in scattered cells of an inflammatory infiltrate of a focal myositis area (arrows). There are no reactions in the sense control. Arrows point at white blood cells. Magnification x315.

**Figure 5:**
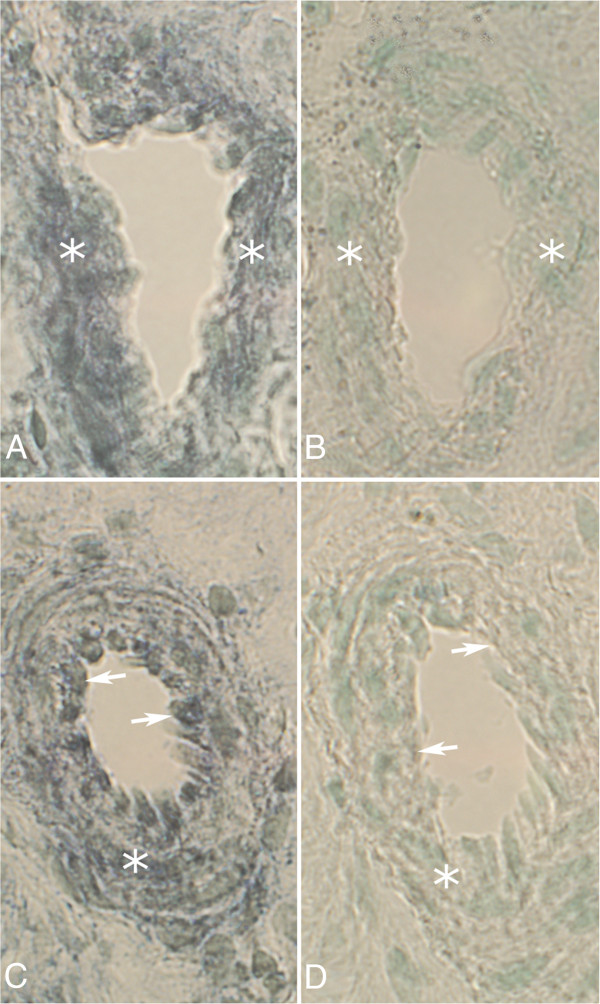
***In situ *hybridization detection of tachykinin mRNA in blood vessel walls.** Blood vessels from focal myositis areas of serial sections from a soleus muscle specimen of the 6w group, non-exercised side (**A**-**D**). *In situ* hybridization (**A**,**C**; antisense staining, **B**,**D**; sense staining) show that expression of tachykinin mRNA is present in the smooth muscle layers (**A**, **C**, asterisks), and also in the endothelial layer (**C**, arrows). There are no reactions in the sense control (**B**, **D**). Magnification x200.

**Figure 6:**
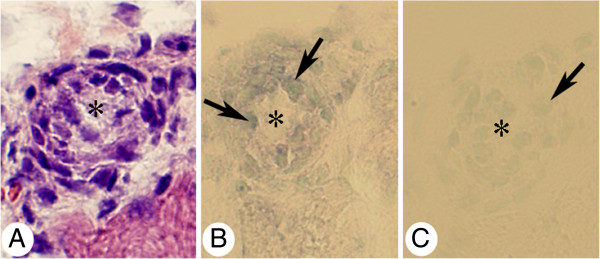
***In situ *hybridization detection of tachykinin mRNA in the wall of a small blood vessel.** Serial sections from a soleus muscle specimen of the 6w group, non-exercised side, showing the presence of tachykinin mRNA expression in cells in the wall of a small blood vessel (**B**) (arrows) (antisense staining), but no reaction in these cells in the sense control (**C**) (arrow). In (**A**), the feature of the blood vessel after staining for H&E is shown. Asterisks in the lumen. Magnification x200.

Expression of tachykinin mRNA was detected as black intracellular reactions in parts of the cells of the inflammatory cell infiltrates of the experimental animals (Figure 3B, 4A). Tachykinin mRNA expression could also be detected within the walls of some of the blood vessels (Figures 5A, C, 6B). Reactions could be seen both in the endothelial part and the smooth muscle part. The most marked reactions were seen for blood vessels of areas exhibiting focal myositis and in nearby located areas. There were no reactions in cells of inflammatory infiltrates and in the blood vessel walls in the sense controls (Figures 3C, 4B, 5B, D, 6C). Importantly, tachykinin mRNA reaction in cells of inflammatory infiltrates and in blood vessel walls were detected in the areas with focal myositis for both the exercised and the non-exercised sides.

### Immunohistochemistry

#### Tachykinin-like immunoreactivity in nerve fascicles and fine nerve fibers

**Figure 7:**
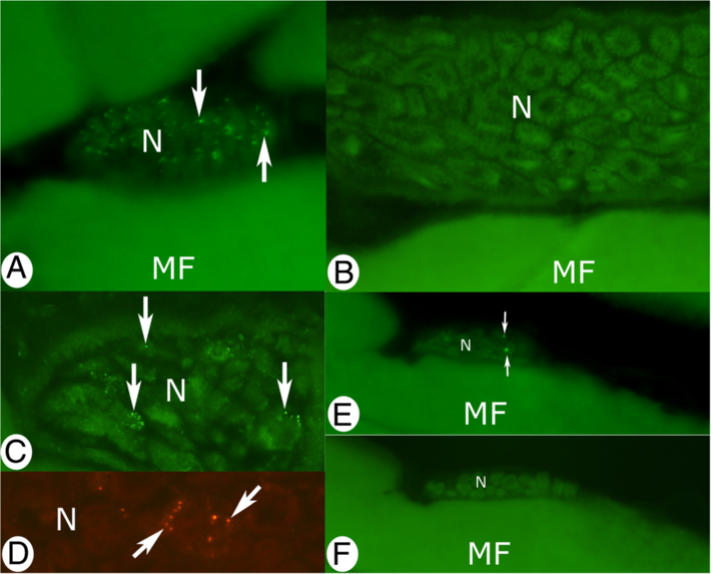
**Tachykinin-like immunoreactivity in nerve fascicles.** Nerve fascicles and parts of nerve fascicles (N) of sections from gastrocnemius (**A**-**C**, **E**, **F**) and soleus (**D**) muscle specimens are shown. The sections were processed for demonstration of tachykinin-like immunoreaction (**A**-**E**). In (**F**) the reaction pattern after preabsorption with SP peptide is shown (the region in F corresponds to the region in **E**). The antibodies applied were sc-14104 (**A**-**C**, **E**) and 8450–0505 (**D**). The specimens were from the control group (**A**, **B**, **E**, **F**) and from the 6w group, non-exercised side, focal myositis area (**C**, **D**). There are immunoreactivities in nerve fascicles in the form of punctuate reactions (arrows **A**, **C**-**E**). These are grouped, as especially seen in (**C**), but also to some extent in (**D**). There are no reactions in the nerve fascicle in (**B**), which is built up of myelinated nerve fibers, and in the control section in (**F**). MF refers to muscle fibers. Magnification x200 (**B**, **C**), x315 (**A**, **D**, **E**, **F**).

**Figure 8:**
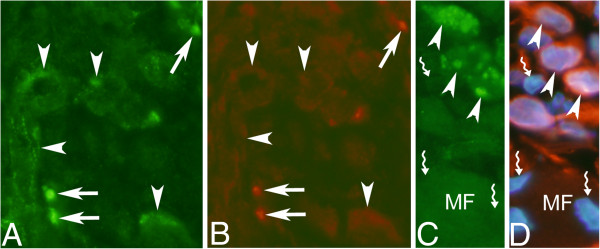
**A,B. Double-staining tachykinin/betaIII-tubulin.** Parts of focal myositis area of experimental animal (1w group, exercised side, gastrocnemius muscle). Staining for tachykinin (**A**) and for betaIII-tubulin (**B**). Tachykinin-like immunoreactivities show co-localization with betaIII-tubulin immunoreactivities (arrows). These reactions are thus nerve fiber related. Tachykinin-like immunoreactivity, but not betaIII-tubulin immunoreactivity, also occur for blood vessel walls (arrowheads). Magnification x200. **c,d. Double-labelling tachykinin/S-100beta.** Parts of focal myositis area of experimental animal (1w group, exercised side, gastrocnemius muscle). Tachykinin-like immunoreactivities occuring in groups are observable (arrowheads, **C**). They are associated with S-100beta immunoreactive cells (arrowheads, **D**). Please note that the nuclei of the cells show a pink/bluish colur reaction (combination of DAPI + S100beta reactions). Nuclei associated with muscle fiber (MF) show the characteristic blue DAPI reaction (curved arrows). Magnification x200.

The great majority of the nerve fascicles of the muscles of both control animals and experimental animals were composed of myelinated fibers and were not showing specific immunoreactivity (Figure 7B). Tachykinin-like immunoreactivity was, however, noted in some of the nerve fascicles of specimens of both groups after stainings for both antibodies applied (Figure 7A, C-E). Nerve fascicles showing these appearances were mainly present in the inflammatory (focal myositis) areas of experimental animals (Figure 7 C,D), but could occasionally be seen in areas with normal morphology (Figure 7A). The immunoreactions occurred as punctuate reactions. The punctuate reactions in nerve fascicles of inflammatory areas were often found to be grouped together (Figure 7C). Such a grouping pattern was not seen for nerve fascicles present in control samples nor in areas with normal morphology of experimental animals. There were also fine tachykinin-like immunoreactivities that were freely dispersed in the inflammatory areas. The reactions were found to co-localize with immunoreactivities for the axonal marker betaIII-tubulin, i.e. they conformed to axonal profiles (Figure 8A,B).

In order to further reveal the characteristics of the nerve-related tachykinin-like immunoreactivities in the myositis areas, double-staining tachykinin/S-100beta was made. This showed that the immunoreactivities usually were located in association with S-100beta immunoreactive cells, i.e. Schwann cells (Figure 8 C,D). The stainings for S-100beta revealed that not only the cytoplasm of these latter cells were immunoreactive, but it also appeared as if their nuclei to a certain extent exhibited S-100beta immunoreactivity. This lead to the occurrence of a pink/bluish colour in these nuclei when DAPI mounting was utilized (combination of S-100beta and DAPI reactions) (Figure 8C,D). This was not the case for nuclei located nearby to the nerve structures (Figure 8C,D) nor in nuclei of nerve fascicles located in areas with normal muscle morphology.

The tachykinin-like immunoreactivity seen in nerve fascicles and in freely dispered axonal profiles was abrogated by preabsorption with SP peptides (peptides from Santa Cruz and Sigma) (Figure 7F).

#### Tachykinin-like immunoreactivity in inflammatory infiltrates

**Figure 9:**
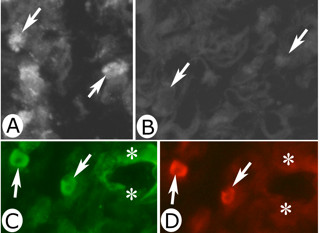
**A,B. Tachykinin-like immunoreactivity in cells in inflammatory infiltrate of myositis area.** Sections of a specimen from a soleus muscle specimen of the 6w group, non-exercised side. In (**A**), the section had been immunostained with 8450–0505, and in (**B**), the section was immunostained with 8450–0505 after preabsorption with tachykinin (SP) (larger area is covered in **B** than in **A**). Immunoreactivity is seen in cells in (**A**), but no reaction is visible in (**B**). Arrows point at cells of inflammatory infiltrate. The immunoreactions show partly a granular appearance (**A**). Magnification x315. **c,d. Immunostaining with polyclonal and monoclonal tachykinin antibodies.** A part of a focal myositis area of a section of a gastrocnemius muscle specimen from the 1w group, exercised side, double-stained for tachykinin using the sc-14104 (**C**) and 8450–0505 (**D**) antibodies, shown at low magnification. Note that the cells of an inflammatory infiltrate are immunolabelled in both (**C**) and (**D**) (arrows), whereas the wall of a small vessel is only immunostained in (**C**) (asterisks). Magnification x315.

Tachykinin-like immunoreactivity was detected in parts of the cells of the inflammatory infiltrates (Figure 9A). This was verified via preabsorption control stainings using SP peptides (Figure 9B) and was seen by use of both tachykinin antibodies used and on both sides. Double-staining showed that reactions for both antibodies were detected in the same cells (Figure 9C,D). The cellular reactions were partly seen as granular reactions (Figure 9A).

**Figure 10:**
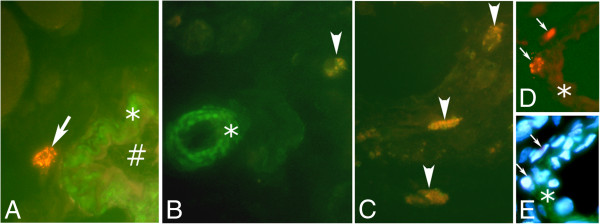
**Double-staining for tachykinin/white blood cell markers.** Sections of soleus muscle tissue of the 6w group, non-exercised side (**A**-**C**) and section of gastrocnemius muscle tissue of the 1w group, exercised side (**D**,**E**). Double stainings for tachykinin (sc-14104) (green) and neutrophil/T-cell marker (MCA805G) (yellowish) are shown in (**A**) and (**D**,**E**). In (**B**), double staining for tachykinin (green) and macrophage marker (CD68) (yellowish) is shown and in (**C**), double staining for tachykinin and eosinophil marker (yellowish) is demonstrated. A white blood cell shows immunoreactivity for the neutrophil/T-cell marker (arrow) but does not show tachykinin-like immunoreactivity (**A**). There is on the other hand tachykinin-like immunoreactivity (green) in the smooth muscle of a vessel wall (asterisk); the sign (#) depicts debris material within the lumen of the blood vessel (**A**). There are unspecific reactions in the endothelium of the vessel wall (**A**). Note that tachykinin-like immunoreactivity (green) can be seen in the macrophage (arrowhead) in (**B**). There is a blood vessel, in which wall there is only tachykinin-like immunoreactivity (asterisk) (**B**). There is also tachykinin-like immunoreactivity in eosinophils (arrowheads) (**C**). Concerning (**D**,**E**): The occurrence of reactions for white blood cells after processing for MCA805G is shown in (**D**) (arrows). The combination of reactions for tachykinin/DAPI is shown in (**E**). The same cells as indicated in (**D**) are indicated in (**E**) (arrows). Note that the cells are not exhibiting tachykinin-like immunoreactivity. There is on the other hand tachykinin-like immunoreactivity in the vessel wall in (**E**) (asterisk). The reaction for MCA805G is masked in (**E**). Magnification x315.

The results of co-localization studies showed that the cells exhibiting expression for T-cells/neutrophil marker (MCA805G) did not exhibit tachykinin-like immunoreactivity (Figure 10A,D,E). Cells expressing reaction for macrophage marker (CD68) showed on the other hand often tachykinin-like immunoreactivity (Figure 10B). Reactions for eosinophil marker (MAB1087) were always seen to co-localize with tachykinin-like immunoreactivity (Figure 10C).

#### Tachykinin-like immunoreactivity in blood vessel walls

Tachykinin-like immunoreactivity was to different extents detected in blood vessel walls when staining with polyclonal antibody sc-14104 was performed, but not after staining with monoclonal antibody 8450–0505 (Figure 9C,D). The immunoreactivity was observed in the walls of both arteries/arterioles and veins/venules.

**Figure 11:**
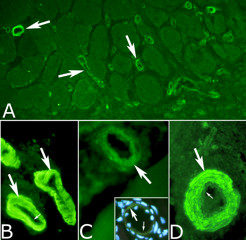
**Tachykinin-like immunoreactivity in blood vessel walls.** Stainings with antibody sc-14104 are shown; gastrocnemius muscle specimens of the 1w group (exercised side) (**A**-**C**, inset **C**), and soleus muscle specimen from 6w (**D**) group (non-exercised side). The region shown in (**A**) is a region located adjacent to a focal myositis area. Large arrows point at blood vessel reactions (**A**-**D**). There are varying degrees of tachykinin-like immunoreactivity in blood vessel walls in (**A**). Vessel walls of the 1w specimens show strong (**B**) (focal myositis area) and moderate (**C**) (vessel in normally appearing area) reactions. A high intensity of immunolabelling is seen in the walls in (**D**). The immunoreactivity is in principle confined to the smooth muscle layers; very weak or neglible reactions are seen in the endothelial layer (small arrows **B**,**D**). In the inset in (**C**), it is obvious that the specific reaction is present in the smooth muscle layer (green colour) (large arrow) whilst the endothelial part is non-reactive (small arrow). In this inset, the DAPI staining of the nuclei is visible. Magnification x50 (**A**), x200 (**B**-**D**).

**Figure 12:**
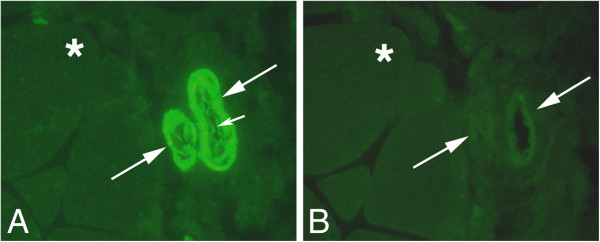
**Tachykinin-like immunoreactivity in arteriolar walls.** Serial sections of a gastrocnemius muscle specimen labelled with antibody sc-14104 (**A**) and for sc-14104 after preabsorption with tachykinin (SP) antigen (**B**). The specimen was from the 3w group and was from the non-exercised side. Note that there is strong immunoreactivity in the arteriolar walls in (**A**), but no reactions in (**B**) (large arrows point at arterioles). The immunoreactivity is mainly detectable in the smooth muscle layer. Note that there is also partially reaction in the endothelial layer (small arrow, **A**). Asterisks indicate similar muscle fibers. The reaction seen in the endothelial part of arteriole in (**B**) is unspecific reaction. Magnification x200.

The blood vessel wall-reactions were seen for both sides of the experimental animals (Figures 10B,E,11). The reactions were abrogated after preabsorption with SP peptide (Figure 12). The immunoreactivities were seen in all groups but were clearly strongest in the 3 and 6 week groups. They could be seen in all parts of the sections but were predominantly seen for blood essels in focal myositis areas and in regions nearby to these. In principle, the walls of all vessels in these areas were immunoreactive (Figure 11A). In specimens of control animals and many of those of 1 week experimental animals (Figure 11C), on the other hand, the blood vessel walls showed generally moderate or weak reactions but could also be seen to be unreactive. Capillaries were always unreactive.

**Figure 13:**
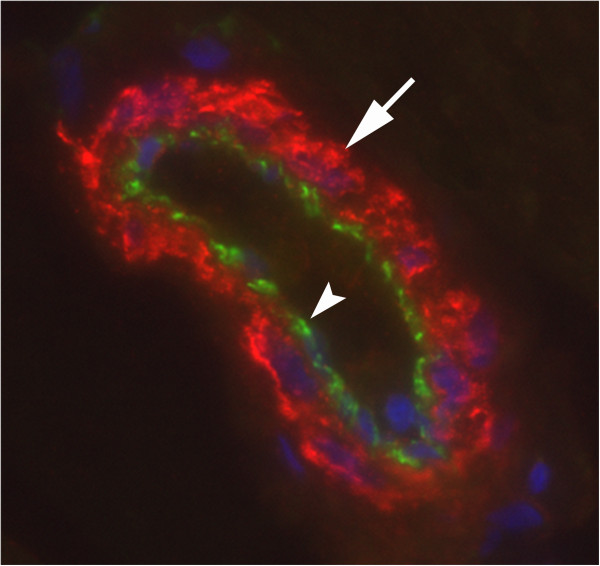
**Double staining CD31/tachykinin.** An arteriole of a specimen from gastrocnemius muscle, 6w group, non-exercised side, is shown. CD31 immunoreactivity is seen in the endothelial part (arrowhead) and tachykinin-like immunoreactivity in the smooth muscle part (arrow). DAPI reaction in nuclei. Magnification x315.

The blood vessel related tachykinin-like immunoreactivity was particularly detected in the smooth muscle layer of the vessels (Figure 11B-D,13) but could sometimes also be seen in the endothelial layer (Figure 12A).

### Tachykinin concentration

**Figure 14:**
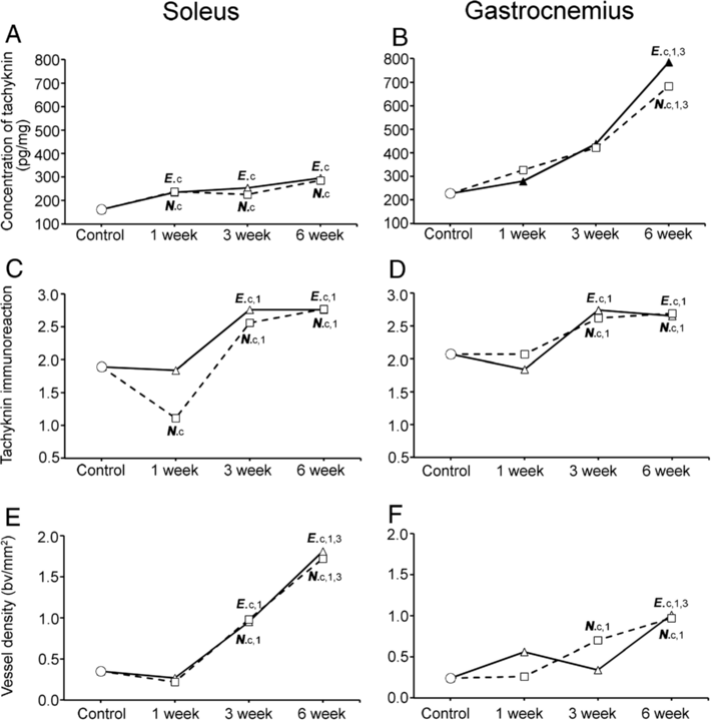
**Overall changes for exercised and non-exercised sides.** Graphs showing the trend of changes in concentration of tachykinin (**A**, **B**), scores of tachykinin-like immunoreaction intensities in blood vessel walls (**C**, **D**) and vessel density (**E**, **F**) in exercised and non-exercised sides of soleus (**A**, **C**, **E**) and gastrocnemius (**B**, **D**, **F**) muscles in the 1w, 3w and 6w groups and controls. Thus, the effects of the exercise upon these parameters for both the experimental (exercised) side and the contralateral (non-exercised) side can here be compared. The situation in the resting (control) situation is also shown. The continuous line shows the situation for the exercised side, and the dotted line shows that for the non-exercised side. E=exercised side, N=non-exercised side. E.c depicts statistical difference as compared to control group, E.1 as compared to 1w group, E.3 as compared to 3w group. N.c describes statistical difference as compared to control group, N.1 as compared to 1w group, N.3 as compared to 3w group.

The tachykinin concentrations for the different stages are described below and are shown in Table 1 and in Figure 14.

**Table 1:** Levels of tachykinin concentrations

**Analysed group**	**Tachykinin (pg/mg)**	
	**Exercised side**	**Non-exercised side**
**Soleus**		
Control	*161± 31*	*161± 31*
1 week	234± 55* (244±556)	237± 33*
3 week	253± 83* (263±896)	225± 30*
6 week	295± 71* (269±628)	285± 54*
**Gastrocnemius**		
Control	*227± 49*	*227± 49*
1 week	*279± 45*	*327± 96 (338±103)*
3 week	*438±77 (422±727)*	*422± 155*
6 week	784± 370*^Δ ▲ ^*(725±462)*	683± 302*^Δ ▲ ^*(683±386)*

Tachykinin concentrations as measured by EIA in the soleus and gastrocnemius muscles after 1w, 3w and 6w of electrical stimulation/exercise and for normal controls are shown. Values are expressed as means and standard deviations. Statistical differences (p < 0.05) between controls and the experiment groups are marked (*), between 1 and 6 week groups (^Δ^) and between 3 week and 6 week groups (^▲^). Values in brackets are the values obtained if the samples for which the CV was comparatively high (>15%) were excluded (in total 9 out of all 83 samples analyzed had >15%).

#### Soleus muscle

##### Exercised side

There was a significant increase in tachykinin concentration in the 1w, 3w and 6w groups as compared to the controls (234 pg/mg vs. 161 pg/mg, p=0.02; 253 pg/mg vs. 161 pg/mg, p=0.004; 295 pg/mg vs. 161 pg/mg, p<0.001, respectively).

##### Non-exercised side

There was also a significant increase in tachykinin concentration in the 1w, 3w and 6w groups as compared to the control group in the non-exercised side (237 pg/mg vs. 161 pg/mg, p<0.02; 225 pg/mg vs. 161 pg/mg, p<0.04; 285 pg/mg vs. 161 pg/mg, p<0.001, respectively).

#### Gastrocnemius muscle

##### Exercised side

The concentration of tachykinin in the 1w group did not differ from that in the control group. There was a tendency of increase in the 3w group, as compared to the concentration seen for the control group (438 pg/mg vs. 227 pg/mg, p=0.055). There was a significant increase in the 6w group as compared to the control group, 1w and 3w groups (784 pg/mg vs. 227 pg/mg, p<0.001; 784 pg/mg vs. 279 pg/mg, p<0.001; 784 pg/mg vs. 438 pg/mg, p=0.002, respectively).

##### Non-exercised side

No statistically significant difference concerning tachykinin concentration was seen between the 1w group and the controls. There was a tendency of an increase in the 3w group as compared to the controls (422 pg/mg vs. 227 pg/mg, p=0.076). A significant increase was, on the other hand, observed in the 6w group as compared to the controls, 1w and 3w groups (683 pg/mg vs. 227 pg/mg, p<0.001; 683 pg/mg vs. 327 pg/mg, p=0.002; 683 pg/mg vs. 422 pg/mg, p=0.019, respectively).

### Assessment of degrees of tachykinin-like immunoreactivity in blood vessel walls

Assessment of the degree of intensity of specific immunoreactivity in blood vessel walls for the different groups was made. The assessment of the entire sections was made, including the possibly occurring myositis areas. The results are summarized below and are shown in Figure 14.

#### Soleus muscle

##### Exercised side

The intensity of immunoreactivity in the 1w group was somewhat lower than that in the control group, but the difference was not statistically significant. The intensity of immunoreactivity was significantly increased in the 3w and 6w groups as compared to the controls (2.76 vs.1.89, p<0.001; 2.76 vs.1.89, p<0.001, respectively) and 1w group (2.76 vs.1.84, p<0.001; 2.76 vs.1.84, p<0.001, respectively).

##### Non-exercised side

The intensity of immunoreactivity was significantly lower in the 1w group than in the control group (1.11 vs.1.89, p=0.001). There was a significant increase in the 3w and 6w groups as compared to the controls (2.56 vs.1.89, p=0.003; 2.77 vs.1.89, p<0.001, respectively). There was also an increase in the 3w and 6w groups as compared to the 1w group (2.56 vs.1.11, p<0.001; 2.77 vs.1.11, p<0.001, respectively).

#### Gastrocnemius muscle

##### Exercised side

The intensity of immunoreactivity in the 1w group was lower than that in the control group, but the difference was not statistically significant. The intensity of immunoreactivity was significantly increased in the 3w and 6w groups compared to the control group (2.74 vs.2.07, p=0.007; 2.65 vs.2.07, p=0.01, respectively). Furthermore, there was also an increase in the 3w and 6w groups as compared to the 1w group (2.74 vs.1.84, p<0.001; 2.65 vs.1.84, p=0.001, respectively).

##### Non-exercised side

The intensity of immunoreactivity was similar in the 1w group and the control group. There was a significant increase in the 3w and 6w groups as compared to the controls (2.62 vs.2.07, p=0.019; 2.69 vs.2.07, p=0.009, respectively). There was also an increase in the 3w and 6w groups as compared to the 1w group (2.62 vs.2.07, p=0.01; 2.69 vs.2.07, p=0.009, respectively).

### Blood vessel density (VD)

The changing pattern concerning the values for blood vessel density (VD) in response to the length of the experiment are shown in Figure 14.

#### Soleus muscle

##### Exercised side

There was no difference in VD between the 1w group and the control group [0.27 blood vessels (bv)/mm^2^ vs. 0.35 bv/mm^2^]. A significantly higher VD was observed in the 3w group as compared to the 1w group (0.95 bv/mm^2^ vs. 0.27 bv/mm^2^, p=0.02) and the controls (0.95 bv/mm^2^ vs. 0.35 bv/mm^2^, p=0.04). In the 6w group, the VD was significantly higher than that in the 1w (1.81 bv/mm^2^ vs. 0.27 bv/mm^2^, p<0.001), 3w (1.81 bv/mm^2^ vs. 0.95 bv/mm^2^, p=0.004) and control (1.81 bv/mm^2^ vs. 0.35 bv/mm^2^, p<0.001) groups.

##### Non-exercised side

The VD in the 1w group did not significantly differ from that in the control group (0.22 bv/mm^2^ vs. 0.35 bv/mm^2^), A significant increase was observed for the 3w group as compared to the 1w (0.98 bv/mm^2^ vs. 0.22 bv/mm^2^, p=0.01) and control (0.98 bv/mm^2^ vs. 0.35 bv/mm^2^, p=0.03) groups. There was also a significant increase in the 6w group as compared to the 1w (1.72 bv/mm^2^ vs. 0.22 bv/mm^2^, p<0.001) and 3 w (1.72 bv/mm^2^ vs. 0.98 bv/mm^2^, p=0.01) groups and the controls (1.72 bv/mm^2^ vs. 0.35 bv/mm^2^, p<0.001).

#### Gastrocnemius muscle

##### Exercised side

There was a tendency of higher VD in the 1w and 3w groups than that in the controls, but the difference was not significant. On the other hand, in the 6w group, there was a significant increase in VD as compared to controls (1.01 bv/mm^2^ vs. 0.24 bv/mm^2^, p<0.001), 1w (1.01 bv/mm^2^ vs. 0.56 bv/mm^2^, p=0.03) and the 3w (1.01 bv/mm^2^ vs. 0.34 bv/mm^2^, p=0.001) groups.

##### Non-exercised side

No statistically significant difference was noted between the 1w group and the controls. However, there was a significant increase in both the 3w and 6w groups as compared to the 1w group (0.70 bv/mm^2^ vs. 0.26 bv/mm^2^, p=0.03; 0.97 bv/mm^2^ vs. 0.26 bv/mm^2^, p=0.001, respectively) and controls (0.70 bv/mm^2^ vs. 0.24 bv/mm^2^, p=0.02; 0.97 bv/mm^2^ vs. 0.24 bv/mm^2^, p<0.001. respectively).

### Comparison between exercised and non-exercised sides concerning tachykinin concentration, intensity of tachykinin-like immunoreactivity in blood vessel walls and VD

There were no significant differences in the mean values for concentration of tachykinin, degrees of tachykinin-like immunoreaction intensity in blood vessel walls and VD between exercised and non-exercised sides in soleus muscle as well as in gastrocnemius muscle after 1w, 3w and 6w of exercise/electrical stimulation.

When the mean values of all experimental animals were grouped together, i.e. after pooling of the soleus and gastrocnemius values, a Pearson correlation test showed that there was a positive and significant correlation between exercised and non-exercised sides concerning tachykinin concentration (r=0.906, p<0.001). It was also found that there was a positive and significant correlation between the two sides concerning intensity of tachykinin-like immunoreactivity in blood vessel walls (r=0.601, p<0.001); Furthermore, there was a significant and positive correlation in VD between the two sides (r=0.642, p<0.001).

Certain correlations between exercised and non-exercised sides were also noted when analyzing for the soleus and gastrocnemius muscles separately (the values for each muscle of all experimental animals, i.e. animals from 1,3 and 6w groups, being grouped together). It was thus found that there was a positive correlation between the two sides concerning tachykinin-like immunoreaction intensity in blood vessel walls (r=0.687, p=0.002) and VD (r=0.809, p<0.001) for the soleus muscle. For the gastrocnemius muscle, a positive correlation between the two sides was noted concerning tachykinin-like immunoreaction intensity in blood vessel walls (r=0.562, p=0.019) and tachykinin concentration (r=0.895, p<0.001).

## Discussion

### Summary of main results

The present study reports the upregulation of tachykinin expression levels in the inflammatory areas and the areas that are adjacent to these in a rabbit experimental model of muscle overuse. Tachykinin-like immunoreactivity was thus frequently observed in axons, and tachykinin mRNA and protein expression was observed in cells of the inflammatory infiltrates. Furthermore, tachykinin-like immunoreactivity and tachykinin mRNA were markedly detected in blood vessel walls in the inflammatory areas. When the degree of tachykinin-like immunoreaction intensity for vessel walls for entire sections was calculated, a significant increase was found to occur for the 3w and 6w animal groups. EIA analyses showed the highest tachykinin concentrations for rabbits at 6w. Concerning the gastrocnemius muscle, the tachykinin levels were significantly higher at 6w compared with the 1w and 3w groups and the control animals. For the soleus muscle, there was a tendency toward an increase in tachykinin concentration between the 3w and 6w stages. Overall, the tachykinin concentration in the muscle tissue increased in a time-dependent manner.

Interestingly, the immunoexpression and mRNA patterns seen for tachykinins and the observations for tachykinin concentration levels and immunoreaction intensity in blood vessel walls noted for the contralateral non-exercised side resembled the observations made ipsilaterally. Thus, we observed positive correlations between exercised and non-exercised sides concerning tachykinin concentration, intensity of tachykinin-like immunoreaction in blood vessel walls and VD.

### Methodological aspects concerning the animal model

In previous structural studies, we noted that the currently used model leads to a marked myositis process, including focal structural changes, and that these changes occur for both the exercised and the non-exercised sides [14]. The aim of the present study was to evaluate the tachykinin reaction pattern and the tachykinin peptide levels in the myositis process.

Our animal model involves the combined effects of unilateral passive flexion/extension of the ankle joint and electrical stimulation of the triceps surae muscle leading to an active contraction in the flexion phase. It is a unique type of model in establishing effects in the muscle, including myositis. The exact relevance of the electrical stimulation in relation to the exercise on the structural changes in the triceps surae muscle is unclear. Nevertheless, in the present study, the model was found to be useful in clarifying the changes that occur concerning the tachykinin system in response to marked overuse. Most importantly, the model was found useful in exploring the contralateral changes that occurred and in clarifying the influence on the various structural elements (white blood cells, blood vessel walls, nerve fascicles) for the tachykinin system in response to marked overuse.

### Methodological aspects concerning stainings

It is noteworthy that our *in situ* hybridization results paralleled those obtained at the protein level by using antibody staining with the polyclonal antibody (sc 14104), i.e. mRNA expression and tachykinin immunoreactivity were noted for white blood cells as well as blood vessel walls. More restricted immunoreactivity (i.e. reactions in white blood cells but not in blood vessel walls) was observed with the monoclonal antibody (8450–0505), which may be related to the fact that this antibody recognizes only the C-terminal end of SP. It is therefore possible that sc 14104, apart from detecting nerve-related as well as white blood cell-related tachykinin, as is also the case for 8450–0505, detects a tachykinin-like peptide variant in the blood vessel walls that is not detected by 8450–0505.

### Increased tachykinin concentration

An increase in staining intensity was observed for the blood vessel walls with antibody 14104 in the 3w and 6w experimental groups. The increase in tachykinin concentration we observed was in principle related to the length of the experiment. It should here be recalled that the magnitude of inflammation increased with the duration of the experiment. Cells in the inflammatory infiltrates exhibited tachykinin-like immunoreactivity. Thus, the increase in tachykinin concentration likely emanates from both the blood vessel walls and the cells of the inflammatory infiltrates. Furthermore, this increase is likely also related to tachykinin expression in nerve fibers, as discussed below.

### Nerve-related tachykinin expression

Tachykinin-like immunoreactivity was noted in only a low number of nerve fascicles of non-inflammatory areas. In accordance with this finding, it is shown that the number of SP-containing C-fibers is much lower in the innervation of muscle than in that of the skin in the rat hindlimb [30]. Overall, only occasional tachykinin-like immunoreactive profiles were observed in nerve fascicles of normal muscle tissue.

On the other hand, frequent fine nerve fibers exhibiting tachykinin-like immunoreaction were observed in inflammatory areas of both sides. The observed increase in tachykinin concentration seen for the experimental animals of especially 6w groups can therefore be related, in part, to frequently occurring nerve fibers showing tachykinin-like immunoreactivity. The immunoreactive nerve fibers were present in nerve fascicles or were freely dispersed in the inflammatory areas. These immunoreactive nerve profiles were usually associated with Schwann cells. As these cells often exhibited not only cytoplasmatic S-100beta reactions, but also signs of nuclear such reactions (as evidenced by pink/bluish nuclear reaction in the S-100beta/DAPI stained sections), it is probable that they represent activated Schwann cells. It is thus known that the S-100beta protein is normally detected in the cytoplasm and at the membranes of the Schwann cells [31], and that Schwann cells are activated after nerve damage, with the S-100beta protein being important for axonal repair [32,33].

In our recent study on the muscular/structural affections of the tissue, abnormal staining patterns for acetylcholinesterase were noted for certain muscle fibers in inflammatory areas [14]. This observation favours that regeneration in response to affection of motor nerves occurs. The results of the present study indicate involvement of tachykinin-like axonal processes, i.e. sensory nerve fibers, in the regenerative processes in the inflammatory areas.

### Tachykinin-like expression in cells of the inflammatory infiltrates

Tachykinin-like immunoreactivity was, by the use of both antibodies utilized, seen in white blood cells in the inflammatory infiltrates. Similarly, tachykinin mRNA reactions were noted for cells of these infiltrates. Using double-staining, this tachykinin-like immunoreactivity was identified in eosinophils and in cells that expressed CD68, but not in cells demarcated by the T-cell/neutrophil antibody. These findings are in accordance with previous observations that SP is detectable in eosinophils [34] and macrophages [35]. Tachykinins have previously been detected in white blood cells in several situations with marked inflammation. There is for example a large number of SP-immunoreactive inflammatory cells in psoriatic plaques [36] and psoriatic skin [37].

Studies suggest that tachykinin produced in non-neuronal cells, such as white blood cells, is of particular importance in pathological conditions [38]. In accordance with this, it has been suggested that tachykinin induction in non-neuronal cells in the respiratory tract of mice after viral infection has important implications for the progression or management of lung disease and infection [39]. Non-neuronal SP is thought to have an important role in psoriasis, via effects on the NK-1 receptor [36]. Remröd and collaborators [37] described that the major part of SP immunoreactivity detected in psoriatic lesions actually was found in inflammatory cells.

It is well-known that tachykinins can have proinflammatory effects in inflammatory conditions [12,13]. It is therefore tempting to suggest that tachykinins have marked proinflammatory effects in the myositic process in this study. It should, however, be recalled that inflammatory-modulating effects of tachykinins may not only be detrimental but also be related to a promoting of survival and cellular immunity [40].

### Tachykinin expression in blood vessel walls

Distinct tachykinin mRNA and tachykinin-like immunoreactions were seen in the blood vessel walls of inflammatory areas and in adjacent regions. The immunoreactions were most clearly observed in the smooth muscle layer, but were also to a lesser extent noted in the endothelial layer. Tachykinin mRNA reactions were seen in both the endothelial and smooth muscle parts. Previous studies have shown that tachykinins can be produced in the endothelium of blood vessels [41], the evidence of which includes observations of the presence of SP at the ultrastructural level [42,43]. Furthermore, tachykinins have also previously been detected in the smooth muscle cells of blood vessel walls, such as those of the human placenta [44]. It has also been found that smooth muscle cells in airways of rats express preprotachykinin and NK-1 receptor mRNA transcripts [45]; this suggests that the smooth muscle cells are influenced via effects on the NK-1 receptors in an autocrine fashion.

### Bilateral effects of the muscle overuse

There were frequent axons displaying tachykinin-like immunoreactions in the focal inflammatory areas of both sides. Tachykinin-like immunoexpression and tachykinin mRNA were noted for cells in the inflammatory infiltrates not only ipsilaterally but also contralaterally. A distinct increase in tachykinin concentration in the muscle tissue, as analyzed via EIA, was obvious on both the right and left sides in late stages of the experiment. There were correlations between the two sides concerning the magnitude of tachykinin-like expressions in blood vessel walls and concerning the tachykinin peptide levels as detected biochemically. All these observations indicate that tachykinins are bilaterally involved in the developing myositis.

Numerous examples in the literature indicate that unilateral inflammation can become coupled to symmetric inflammation in the contralateral side, possibly involving a neurogenic component [46,47]. In patients with rheumatoid arthritis, a symmetrical spread of the inflammatory response is thought to stem from increases in sensory neural activity on the contralateral side [48]. Accordingly, it has been hypothesized that the presumable neurogenic component in inflammatory arthritis, giving rise to symmetrical joint involvements, is related to primary-afferent activation leading to contralateral activation of homologous afferents [49]. This might represent a self-protective mechanism that serves to prime the contralateral joint following an ipsilateral involvement, the protective response in the long run, however, becoming destructive [50]. It is possible that contralateral effects may not only exist in the overlapping terminals of afferents but also in motor neuron dendrites and that contralateral effects mediated by spinal interneurones may also exist [50]. There is also evidence which favours that unilateral strength training can increase the capacity of the motor cortex to drive the homologous, untrained muscles on the contralateral side [51]. Unilateral peripheral burn injury has been shown to lead to long-lasting allodynia that can spread to the contralateral limb, with hyperexcitability in the dorsal horn and microglial activation in both ipsilateral and contralateral sides of the spinal cord [52].

Previous findings concerning the contralateral responses to unilateral inflammatory lesions and their relationship to neurological mechanisms have been extensively reviewed by Schenker and collaborators [53]. Of special interest for the present study, is the finding that electrical stimulation can lead to orthodromic activation of afferent nerve fibers which can in turn lead to release of SP and changes in both the stimulated and contralateral muscles [54]. Therefore, electrical stimulation may not only lead to an increased muscle activity but also an orthodromic activation of afferent nerve fibers [54].

Tachykinins can be important in the formation of bilateral effects after a unilateral inflammatory stimulus. Following induction of monoarthritis with complete Freund´s adjuvant, one study showed that arthritis with cartilage degeneration occurred on both sides of the animal and that it could be ameliorated by injections of NK-1 receptor antagonists intrathecially prior to arthritis induction [55]. Bilateral effects concerning the tachykinin system have been observed following unilateral formaldehyde injections [56], heat injury [57] and craniofacial inflammation [58]. Furthermore, previous work using the current model and focusing on the Achilles tendon showed that tendinosis-like features were observed not only in the exercised tendon but also in the tendon in the contralateral non-exercised side following unilateral overuse [19]. An important observation in the present study is that besides the occurrence of frequent axons showing tachykinin-like immunoactivity in the inflammatory areas bilaterally, a bilateral up-regulation of the tachykinin system was found for the blood vessel walls. Furthermore, tachykinin-like immunoreactivity occurred for white blood cells. This means that the tachykinin system operates not only via tachykinin from the nerve fibers, but also via tachykinin from white blood cells and blood vessel walls, to bring about influences in the tissue.

A drawback with the setup of the study was the fact that other muscles in the contralateral side than the triceps surae muscle were not collected and examined so that changes in the general circulation could be considered. However, the types of nerve changes we observed should not depend on circulatory effects. Furthermore, the tissue changes we noted were not generalized but focal, with certain parts of the muscles retaining a normal morphology (c.f. Figure 1).

In accordance with an assumption that the contralateral effects are related to influences on the nerves, is the previous notion that unilateral nerve injury can lead to bilateral allodynia [59] and that there exists a “mirror-image” pain implicating the presence of a signalling system between the two sides [60]. In addition, lesions to the nociceptive nerves supplying the ipsilateral or contralateral limb prior to an inflammatory insult, can abolish the contralateral response [53]. Thus, the existence of central mechanisms is clearly more probable than mechanisms via circulating factors [50].

Here we should stress, however, that we neither assessed the nervous system from a functional point of view, nor evaluated the structural characteristics at the levels of the dorsal root ganglia and spinal cord. Studies on these aspects should be performed in the future. It seems nevertheless likely that primary-afferent activations may be involved in the initiation of the bilateral effects.

### Functional aspects: final comments in relation to the NK-1 receptor

Tachykinins may be involved in pro-inflammatory [12,13] as well as repair processes [61,62] in the myositis and muscular derangement situation. Overall, tachykinins can hereby operate in an autocrine/paracrine fashion [43,63,64]. In accordance with this suggestion, we have noted that white blood cells as well as blood vessel walls and nerve profiles in the inflammatory areas of the animals here studied showed positive immunoexpression for the NK-1 receptor [18]. Using double-stainings, we noticed a co-localization between tachykinin and NK-1 receptor in some of the white blood cells and some of the axonal nerve profiles [18].

In previous studies focusing on the Achilles tendon and utilizing the rabbit overuse model, we have observed that local administration of tachykinin (SP) outside the Achilles tendon accelerates hypercellularity and angiogenesis for this tendon, suggesting that tachykinins are also involved in tendinosis (tendinopathy) development in this model [65].

The bilateral upregulation of tachykinin immunoreactivity and increase in tachykinin concentration may be linked to the bilateral up-regulation in the NK-1 R reactions [18]. Further studies should evaluate whether treatments that favour tachykinin effects or block its effects can be useful in situations with myositis. It has long been considered that NK-1 receptor blocking treatments might be useful in inflammatory situations [66], hereby suppressing pro-inflammatory cytokine responses [67,68]. However, as described above, effects of tachykinins are not only related to pro-inflammatory influences but also trophic and healing influences. Thus, tachykinins have double-edged effects [69] that need to be considered.

## Conclusions

In conclusion, we show that the tachykinin system is up-regulated both in the exercised/electrically stimulated side and in the contralateral non-experimental side in the currently used rabbit model of muscle overuse. Thus, interestingly, the upregulation of the tachykinin system occurs bilaterally. This was related to an increase in tachykinin expression in the nerves and the blood vessel walls of the inflammatory areas and to the presence of tachykinin expression in the infiltrating white blood cells. The tachykinin upregulation was visualized via immunohistochemical, *in situ* hybridization and EIA analyses. Tachykinins thus appear to be important in the processes that occur in the myositis and muscle derangement following muscle overuse. The results show that our animal model can be useful in further studies evaluating the involvement of contralateral muscles/innervations in response to unilateral marked overuse.

## Competing interests

There are no competing interests.

## Authors’ contributions

SF, PS and JY made the initial conceiving and design of the animal experiments. YS performed the experimental stainings and EIA analyses. YS and SF performed the microscopic analyses. YS, SF and PS evaluated the results. SF, YS and PS were dealing with the writing process, SF hereby being responsible. YS, SF, PS and JY all approved the final version of the manuscript.

## Pre-publication history

The pre-publication history for this paper can be accessed here:

http://www.biomedcentral.com/1471-2474/14/134/prepub
